# PEGylation of the antimicrobial peptide LyeTx I-b maintains structure-related biological properties and improves selectivity

**DOI:** 10.3389/fmolb.2022.1001508

**Published:** 2022-10-13

**Authors:** Júlio César Moreira Brito, Lucas Raposo Carvalho, Amanda Neves de Souza, Guilherme Carneiro, Paula Prazeres Magalhães, Luiz Macêdo Farias, Natália Rocha Guimarães, Rodrigo Moreira Verly, Jarbas Magalhães Resende, Maria Elena de Lima

**Affiliations:** ^1^ Fundação Ezequiel Dias, Diretoria de Pesquisa e Desenvolvimento, Belo Horizonte, MG, Brazil; ^2^ Departamento de Química, Instituto de Ciências Exatas, Universidade Federal de Minas Gerais, Belo Horizonte, MG, Brazil; ^3^ Departamento de Química, Instituto de Ciências Exatas, Universidade Federal dos Vales do Jequitinhonha e Mucuri, Diamantina, MG, Brazil; ^4^ Departamento de Farmácia, Faculdade de Ciências Biológicas e da Saúde, Universidade Federal dos Vales do Jequitinhonha e Mucuri, Diamantina, MG, Brazil; ^5^ Departamento de Microbiologia, Instituto de Ciências Biológicas, Universidade Federal de Minas Gerais, Belo Horizonte, MG, Brazil; ^6^ Programa de Pós-graduação em Medicina e Biomedicina da Santa Casa de Belo Horizonte, Belo Horizonte, MG, Brazil

**Keywords:** PEGylation, antimicrobial peptide, LyeTx I-b, structure, post-translational modification, peptide-membrane interaction

## Abstract

The biological activity of antimicrobial peptides and proteins is closely related to their structural aspects and is sensitive to certain post-translational modifications such as glycosylation, lipidation and PEGylation. However, PEGylation of protein and peptide drugs has expanded in recent years due to the reduction of their toxicity. Due to their size, the PEGylation process can either preserve or compromise the overall structure of these biopolymers and their biological properties. The antimicrobial peptide LyeTx I-b_cys_ was synthesized by Fmoc strategy and coupled to polyethylene glycol 2.0 kDa. The conjugates were purified by HPLC and characterized by MALDI-ToF-MS analysis. Microbiological assays with LyeTx I-b_cys_ and LyeTx I-bPEG were performed against *Staphylococcus aureus* (ATCC 33591) and *Escherichia coli* (ATCC 25922) in liquid medium. MIC values of 2.0 and 1.0 µM for LyeTx I-b_cys_ and 8.0 and 4.0 µM for LyeTx I-bPEG were observed against *S. aureus* and *E. coli*, respectively. PEGylation of LyeTx I-b_cys_ (LyeTx I-bPEG) decreased the cytotoxicity determined by MTT method for VERO cells compared to the non-PEGylated peptide. In addition, structural and biophysical studies were performed to evaluate the effects of PEGylation on the nature of peptide-membrane interactions. Surface Plasmon Resonance experiments showed that LyeTx I-b binds to anionic membranes with an association constant twice higher than the PEGylated form. The three-dimensional NMR structures of LyeTx I-b_cys_ and LyeTx I-bPEG were determined and compared with the LyeTx I-b structure, and the hydrodynamic diameter and zeta potential of POPC:POPG vesicles were similar upon the addition of both peptides. The mPEG-MAL conjugation of LyeTx I-b_cys_ gave epimers, and it, together with LyeTx I-bPEG, showed clear α-helical profiles. While LyeTx I-b_cys_ showed no significant change in amphipathicity compared to LyeTx I-b, LyeTx I-bPEG was found to have a slightly less clear separation between hydrophilic and hydrophobic faces. However, the similar conformational freedom of LyeTx I-b and LyeTx I-bPEG suggests that PEGylation does not cause significant structural changes. Overall, our structural and biophysical studies indicate that the PEGylation does not alter the mode of peptide interaction and maintains antimicrobial activity while minimizing tissue toxicity, which confirmed previous results obtained *in vivo*. Interestingly, significantly improved proteolytic resistance to trypsin and proteinase K was observed after PEGylation.

## 1 Introduction

Infections caused by antibiotic-resistant bacteria, also known as superbugs, are responsible for the deaths of more than 700,000 people per year worldwide and, if left unchecked, the number of deaths may rise by more than 10 million by 2050, surpassing chronic diseases like diabetes and cancer (Burki, 2018). In this context, the search for novel antimicrobial agents against superbugs is an urgent need, and antimicrobial peptides (AMPs) stand out from their conventional counterparts due to their bactericidal potential, anti-inflammatory activity, and modulation of the immune system (Kang et al., 2012; Fosgerau and Hoffmann, 2015; Zhang and Gallo, 2018; Chen and Lu, 2020). Fungal and bacterial cell membrane phospholipids are known targets for these biopolymers, which act by increasing permeability, leading to leakage of intracellular material and subsequent death of the microorganism (Brito et al., 2021; Reis et al., 2021).

Common properties of AMPs include relatively small size, clear amphipathic character, and net positive charge, typically in the range of + 2 to + 9, represented by an accumulation of His and/or Lys residues (Hancock and Sahl, 2006; Wang et al., 2017; Lei et al., 2019). Considering the typical p*K*_a_ range of the histidine side chain, the imidazole rings are usually partially protonated (Resende et al., 2008; Tu et al., 2009; Nunes et al., 2021). Although AMPs have interesting properties for biological applications, problems such as low bioavailability and rapid degradation hinders their widespread use. Fortunately, post-translational modifications are useful strategies to counteract these drawbacks, namely glycosylation, lipidation, and PEGylation (Grimsey et al., 2020; Li et al., 2021; Rezende et al., 2021).

Since structural parameters and biological activity are closely related aspects of AMPs, it is interesting to ensure that these post-translational modifications, especially PEGylation, improve antimicrobial properties without affecting their overall structure (Lawrence and Price, 2016). Although there are some reports of three-dimensional structures of PEGylated peptides and proteins (Digilio et al., 2003; Gardiner et al., 2009; Cattani et al., 2015), their antimicrobial representatives have received less attention (Ilyas et al., 2021). This lack of representation suggests that the PEGylation of AMPs is an interesting topic to approach and discuss, given the paucity of data that can explicitly show whether PEGylation results in substantial conformational changes of AMPs.

LyeTx I-b is a 24 residue-long peptide derived from LyeTx I, isolated from the spider venom of *Lycosa erythrognata* (Santos et al., 2010; Reis et al., 2018; Melo-Braga et al., 2020). It is a potent broad-spectrum antimicrobial agent (Reis et al., 2018; Brito et al., 2021) and its PEGylated derivative, LyeTx I-bPEG, shows activity against *Acinetobacter baumannii* both *in vivo* and *in vitro* (Brito et al., 2021).

The aim of this work is to demonstrate the antimicrobial activity of the peptide LyeTx I-bPEG in Gram-positive and negative multidrug-resistant bacteria, *in vitro*, evaluate its cytotoxicity against VERO cells, and correlate this data with structural and biophysical parameters to better understand the role of PEGylation in this AMP.

## 2 Materials and methods

### 2.1 Materials and microorganisms

Fmoc-protected L-amino acid derivatives and Fmoc resin (Rink amide 0.52 mmol · g^−1^) were purchased from *Iris Biotech Gmbh* (Marktredwitz, Germany). 10 ml low-density polyethylene (LDPE) syringes, sodium chloride (NaCl), dichloromethane (DCM), dimethylformamide (DMF), diisopropyl ether, ethanol, monobasic sodium phosphate, and isopropanol (IPA) were purchased from Química Moderna (Sao Paulo, Brazil). Acetic anhydride, 3-(4,5-dimethyl-2-thiazolyl)-2,5-diphenyl-2*H*-tetrazolium bromide (MTT), potassium cyanide (KCN), diisopropylcarbodiimide (DIC), sodium dodecyl sulphate (SDS), Dulbecco’s modified Eagle medium (DMEM), phenol, glycerol, 1-hydroxybenzotriazole (HOBt), α-cyano-4-hydroxycynnamic acid (α-cyano), ninhydrin, 4-methylpiperidine (PIPE), pyridine, triisopropylsilane (TIS), *tris*(2-carboxyethyl)phosphine (TCEP) and Triton X-100 were purchased from Sigma (Saint Louis, MO, United States). Methyl-PEG-maleimide (mPEG-MAL) was acquired from Polysciences Inc. (Warrington, United States). Acetonitrile and trifluoroacetic acid (TFA) were purchased from J. T. Baker (Center Valley, PA, United States). Peptide calibration standard II was purchased from Bruker Daltonics (Hamburg, Germany). Difco™ Tryptic Soy Agar (TSA) and Difco™ Mueller Hinton Broth (MH) were purchased from BD (Sparks, MD, United States).

*Escherichia coli* (ATCC 25922), *Staphylococcus aureus* (ATCC 33591), and three multidrug-resistant isolates (two *E. coli* and one *S. aureus*) were provided by Laboratory of Oral Microbiology and Anaerobes, Department of Microbiology, Institute of Biological Sciences, Federal University of Minas Gerais. Renal epithelial cells of the African green monkey (*Cercopithecus aethiops*) (VERO, ATCC CCL-81) were provided by the Federal University of Sao Joao del-Rei (UFSJ).

### 2.2 Synthesis of peptides LyeTx I-b and LyeTx I-b_cys_

Peptide synthesis was performed according to an adapted Fmoc protocol (Hansen and Oddo, 2015; Behrendt et al., 2016), described as follows: 160 mg of Rink amide resin was weighed and placed in a 10 mL LDPE syringe previously quilted with a polyurethane filter. The system was washed with DCM and IPA, deprotected (i.e*.*, the Fmoc portion was removed) with a 20% PIPE/DMF (v/v) solution and stirred for 15 min at 240 rpm in a shaker table (Fanem, Sao Paulo, Brazil). The resin was washed again with DCM and IPA and received a solution containing the respective amino acid derivative (4 molar equivalents), 1.5 mL of DCM, 1.5 mL of distilled DMF, 56 mg of HOBt, and 57 µL of DIC, and the system was stirred (240 rpm) for 2 hours. After coupling the last amino acid residue, the peptidyl-resin was deprotected and acylated by adding 2 mL DMF and 30 µL acetic anhydride, and the system was then stirred for 2 hours. For cleavage, the peptidyl-resin was dried under N_2_ and weighed. An acid cocktail (94% TFA, 2.5% H_2_O, 1.0% TIS, and 2.5% v/v EDT) was prepared at a ratio of 8.0 ml of solution per Gram of dry peptidyl-resin. The cocktail was aspirated into the syringe, and it was then stirred (240 rpm) for 3 hours. The acid solution was then transferred to a centrifuge tube and the syringe was washed twice with TFA (1.0 mL).

The acid solution was dried under N_2_ flux, and the product was precipitated with cold diisopropyl ether (5.0 mL) and centrifugation at 4000 rpm in a Thermo Fisher Scientific centrifuge (Osterode Am Harz, Germany) for 5 min and then lyophilized. The deprotection, coupling, and acylation reactions were monitored using the Kaiser test (Hansen and Oddo, 2015; Behrendt et al., 2016).

### 2.3 Purification of peptides and conjugation of LyeTx I-b_cys_ with mPEG-MAL

The synthesized product was purified by RP-HPLC using a Shimadzu LC20AD/SPDM20A/RID20 (Kyoto, Japan) with a Phenomenex C18, 250 mm, 10 mm semi-preparative column equilibrated with nine parts TFA in water 0.1% (v/v, phase A) 90%, and one part TFA 0.08% in acetonitrile (v/v, phase B) 20%, using a wavelength of 220 nm at the detector. The gradient of phase B was linear as follows: 10% to 5 min, increasing to 60% from 5 to 30 min, increasing to 100% from 30 to 45 min and remaining at this percentage until 50 min, and returning to 10% from 50 to 60 min.

**FIGURE 1:**
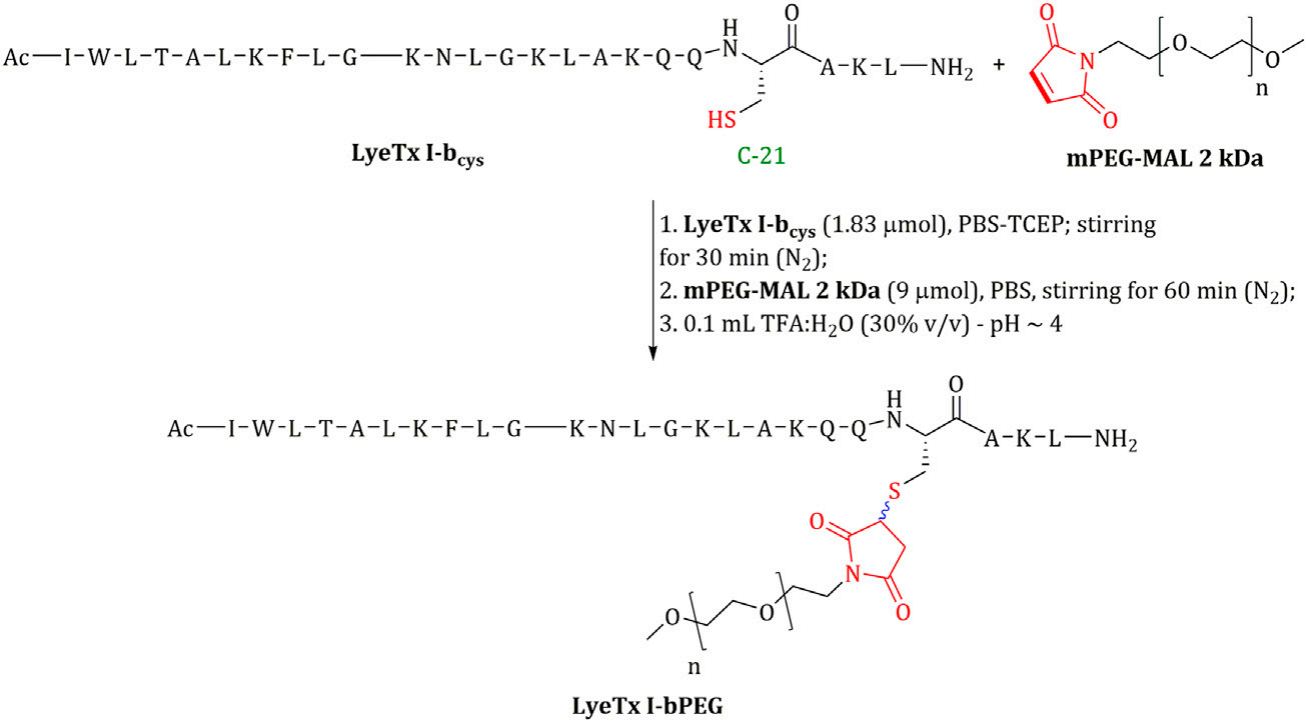
Schematic presentation of the conjugation between LyeTx I-b_cys_ and mPEG-MAL to afford LyeTx I-bPEG. Directly involved groups are shown in red.

Conjugation with mPEG-MAL, using the reagents shown in Figure 1, was performed according to Burg et al. (2007); Brito et al. (2021), and the product was purified according to Brito et al. (2021). Reaction yield was over 80% and purification yield was over 90%.

### 2.4 Characterization by MALDI-TOF

The fractions of chromatographic peaks with the largest absorbance were collected and analyzed by Matrix-Assisted Laser Desorption Ionization–Time of Flight–Mass Spectrometry (MALDI-ToF-MS) using an Auto Flex III (Bruker Daltonics, Hamburg, Germany) mass spectrometer. In summary, samples were placed in a Bruker Daltonics MTP Anchorchip 384 BC (Hamburg, Germany), mixed with a saturated α-cyano solution, and dried at room temperature. Samples and standards were analyzed in the spectrometer using the Pepmix (up to 4 kDa) or the Protomix (4 kDa to 8 kDa) methods (Strohalm et al., 2008).

Mass spectra (MS) were recorded in positive mode, after calibration and using the peptide calibration standard. Mass spectrometry was also used to sequence the peptide LyeTx I-b_cys_ using MS/MS. Spectra were generated and analyzed using mMass Data Miner software (Strohalm et al., 2008). MALDI-ToF analyzes were performed at the Centro de Laboratórios Multiusuários (CELAM) (ICB/UFMG).

### 2.5 Antimicrobial activity: Minimum inhibitory concentration evaluation

In order to determine MIC values of the PEGylated and the non-PEGylated peptides, bacterial isolates were cultured in TSA. After incubation for 24 h, at 37°C, under aerobic conditions, colonies were collected using an inoculation loop and transferred to saline solution (0.9% m/v NaCl). The inoculums were checked in a spectrophotometer at 600 nm; colonies were added until an absorbance corresponding to 1.5 × 10^8^ cells · mL^−1^ was obtained. Then, a 150-fold dilution was performed to achieve a concentration of 1 × 10^6^ cells · mL^−1^. MIC assays were performed in microdilution plates employing inoculums of 10^4^ bacterial cells per well and concentrations of peptides ranging from 32 to 0.25 μM. All analyses were performed in triplicate. Positive (MH + bacterial inoculum) and negative (MH and MH + saline solution) controls were included in each batch of tests.

The specific values of molar peptide concentration used in this work were estimated according to the method described by Schmid (2001).

### 2.6 *In vitro* cytotoxicity evaluation

*In vitro* toxicity to renal epithelial cells of *Cercopithecus aethiops* (VERO ATCC CCL-81) was evaluated using 3-(4,5-dimethylthiazol-2-il)-2-5-dipheniltetrazolium (MTT; Sigma Aldrich, St. Louis, MO, United States) and the colorimetric method (Mosmann, 1983). In summary, cells were grown, density of 2.5-3.0 × 10^4^ cells per well, in a 96-well tissue culture plate (CytoOne, CC7682-7596) in DMEM supplemented with 5% fetal bovine serum, 50 mg · mL^−1^ l-glutamine, and 0.3% of an antimicrobial solution (penicillin-streptomycin-amphotericin B; 10,000 U · mL^−1^ + 10 mg · mL^−1^ + 2.0 mg · mL^−1^). This plate was then incubated for 24 h at 37°C in a 5% CO_2_ atmosphere, and the cells were treated with the peptides at various concentrations for 24 h. After incubation, the cells were washed and incubated with 100 µL DMEM containing 20 µL MTT for 4 h at 37°C. Absorbance measurements were performed using a microplate reader (Molecular Devices, Sunnyvale, CA, United States) at 540 nm and cytotoxic concentrations for 50% of the cells (CC_50_) were calculated by linear regression analysis.

### 2.7 Selectivity index

To verify the selectivity of the compound for the tested pathogen compared to VERO cells, the selectivity index (SI) was calculated as described by Lyu et al. (2016). To calculate the index, the obtained IC_50_ values for each bacterial isolate tested were divided by the CC_50_ value obtained for VERO cells.

### 2.8 Preparation of large unilamellar vesicles

LUVs were prepared using the phospholipids 1-palmitoyl-2-oleoyl-*sn*-glycero-3-phosphocholine (POPC) and 1-palmitoyl-2-oleoyl-*sn*-glycero-3-phospho-(1′-*rac*-glycerol) (POPG) for Surface Plasmon Resonance Spectroscopy (SPR), Dynamic Light Scattering (DLS), and zeta potential (*ζ*) measurements. The appropriate amount of phospholipid in a molar ratio of POPC:POPG of 3:1 was transferred to a glass tube and suspended with 2 ml of chloroform at room temperature. The organic solvent was removed in a rotary evaporator to form a lipid film, which was hydrated with 2 ml of 10 mM Tris-HCl buffer (pH 8.0) containing 100 mM NaCl at 35°C to give a 10 mM stock solution. The resulting multilamellar vesicles (MLVs) were subjected to five freeze/thaw cycles using liquid nitrogen and a water bath at 35°C (five cycles). The MLVs were extruded in a 10 ml stainless steel extruder (Lipex Biomembranes Inc. Vancouver, Canada) at almost 35°C to obtain LUVs with size of 100 nm. The total lipid concentration of the extruded LUVs was estimated by a colorimetric method as described by Stewart (1980).

### 2.9 Dynamic light scattering and zeta potential (*ζ*-potential)

The changes in hydrodynamic diameter (*D*_h_) and zeta potential (ζ-potential) of POPC and POPC:POPG LUVs during titration with the peptide solutions were measured at 25°C using a Z98 Zetasizer Nano ZS Malvern™ model BI-900 particle analyzer (Worcestershire, United Kingdom). The light intensity of the monochromatic laser (4 mW Ne laser, λ of 633 nm) scattered was detected at a 173° angle. Experiments were performed in triplicate by titrating each peptide at 8.0 mM in 500 μM POPC:POPG LUVs, both suspended in 10.0 mM Tris-HCl buffer, pH 8.0. Experiments consisted of ten consecutive injections of 1.0–10.0 µL of peptide solution into the cuvette (1.0 mL) containing 700 μL of the vesicle solution. After each injection, an interval of 20 min was allowed for the system to stabilize before the hydrodynamic diameter and ζ-potential were measured.

### 2.10 Calcein leakage studies

The membrane permeation capabilities of LyeTx I-b and LyeTx I-bPEG was evaluated at 25°C by measuring calcein leakage from LUVs using a Cary Eclipse Fluorescence Spectrophotometer (Agilent, Palo Alto, United States) with excitation wavelength at 490 nm and emission wavelength at 515 nm. To acquire data, 250 µL of POPC:POPG 3:1 LUVs (0.25 mM) were added to a fluorescence cuvette containing Tris-HCl buffer (pH 7). The increase of calcein fluorescence as a function of time was measured continuously after the addition of different masses of the peptides (0.5, 1, 2, 4, 8, and 12 µg for both) to the samples with final volume of 2.5 mL.

After 15 min, 10 µL of 10% (v/v) Triton X-100 solution was added to the cuvette to attain complete vesicle leakage and maximum calcein fluorescence. The percentage of calcein leakage was calculated according to Eq. 1,

$$\%\;Leakage=\frac{I_{0}-I_{t}}{I_{0}-I_{T}}\times 100$$ (1)

where *I*_**0**_ is the fluorescence prior to peptide addition, *I*_**t**_ is the measured time-dependent fluorescence after peptide addition and *I*_**T**_, the fluorescence after addition Triton X-100.

### 2.11 Surface plasmon resonance

For information about peptide-membrane interactions, SPR measurements were performed at 25°C at a flux of 10 μL · min^−1^ for LUV immobilization and recorded at 850 nm using an SPR Navi™ 200 instrument (BioNavis Ltd. Ylöjärvi, Finland). Measurements were performed in angular scan mode (40 – 78°), with SPR curves recorded every 3.5 s. SPR gold sensor chips were previously functionalized with DL-dithiothreitol (DTT) as described elsewhere (DazaMillone et al., 2018). DTT-gold chips were used for phospholipid immobilization, washed with successive 5 min injections (50 μM · min^−1^) of 5% Hellmanex™ III (Sigma, St Louis, MO), 2-propanol, and Milli-Q water, *in situ* in the flow channel, immediately before each experiment. For each measurement, the sensor chip was first exposed to the running buffer (10 mM Tris-HCl buffer, pH 8.0, 50 μM · min^−1^) and then 50—250 μM POPC:POPG LUVs for approximately 10—12 min (10 μM · min^−1^) until the baseline stabilized. Experiments consisted of 50 µL injections of 10 µM peptide solutions in running buffer. The surface partition coefficient (*K*) of peptide-membrane interactions was determined from SPR experiments by fitting the data to the following equation, taking into account the electrostatic interaction (Bong et al., 2000; Muñoz-López et al., 2021):

$$\Delta RU_{eq}=\Delta RU_{eq\;\left(\max\right)}=\left[\frac{K\cdot c_{L}}{\exp\left(\frac{Z_{p}\Psi F}{RT}\right)+K\cdot c_{L}}\right]$$ (2)

Where Δ*RU*_eq_ is the change in observable *RU*_eq_ intensity 15 min after peptide injection, Δ*RU*_eq(max)_ is the maximum change in observable *RU*_eq_ obtained by fitting the raw data, *Z*_*p*_ is the charge on the peptide, Ψ is the membrane surface potential, *F* is Faraday’s constant, *R* is the universal gas constant, and *T* is temperature. The concentration of accessible lipid (considering the outer leaflet of the membrane bilayer, which accounts for 60% of the total lipid concentration) is represented by *c*_*L*_. Quantification of the total phospholipid content immobilized in the sensor ship was performed as described by Junior et al. (2017).

The membrane surface potential, Ψ, was calculated from the ζ-potential and considering the exponential decay of the electrostatic potential (Verwey et al., 1948; Tadros, 2013), according to the following formula:

$$\Psi =\zeta e^{\kappa x}$$ (3)

Here, *ζ* stands for the ζ -potential, κ for the inverse of the Debye length, and *x* for the hydrodynamic layer thickness, which in this case is assumed to be *x* = 0.25 nm from the POPC:POPG LUV surface (Satoh, 1995; Chibowski and Szcześ, 2016). The *ζ* -potentials of 0.5 mM POPC:POPG LUVs were measured at 25°C in a Malvern Zetasizer Nano ZS™ particle analyzer (Malvern Instrument Ltd. Worcestershire, United Kingdom) as described by Junior et al. (2017). The *ζ* -potential value used in Eq. 3 (−40.5 ± 0.1 mV) corresponds to the average of the values of three LUV solutions.

### 2.12 Nuclear magnetic resonance spectroscopy, data analysis and structure calculations

Two-dimensional NMR experiments were performed for LyeTx I-b_cys_ and LyeTx I-bPEG in order to determine their three-dimensional structures. Experiments have been carried out for LyeTx I-b_cys_ at 2.0 mM and for LyeTx I-bPEG at 1.5 mM in TFE-*d*_2_:H_2_O (60:40, v:v) solutions (600 μL) adjusted to pH 7.0 using phosphate buffer at 20 mM. 2,2-diMethyl-2-silapentane sulfonate (DSS) at 1.0 mM was used as the internal reference for LyeTx I-b_cys_ and at 2.0 mM for LyeTx I-bPEG. TOCSY, NOESY and HSQC data acquisition was done at 20°C on a Bruker Avance Neo 600 spectrometer equipped with a 5 mm BBO multinuclear SmartProbe. ^1^H NMR data was acquired for LyeTx I-b_cys_ using a spectral width of 6578 Hz and for LyeTx I-bPEG with a spectral width of 6849 Hz. Water suppression was achieved by pre-saturation.

Total Correlation SpectroscopY (TOCSY) experiments were made using the DIPSI-2 scheme (Rucker and Shaka, 1989). The parameters used for the experiments were: spectral width of 6578 Hz, 512 *t*_1_ increments with 16 transients of 4096 points for LyeTx I-b_cys_ and spectral width of 6849 Hz, 512 *t*_1_ increments with 16 transients of 4096 points for LyeTx I-bPEG. A spin-lock time of 80 ms was used in the TOCSY experiments. Nuclear Overhauser Effect SpectroscopY (NOESY) spectra (Kumar et al., 1980) were acquired with mixing times of 100, 150, 200 and 250 ms for LyeTx I-b_cys_ and of 100, 120, 150 and 250 ms for LyeTx I-bPEG in order to check whether spin diffusion would ensue. The parameters used for NOESY experiments were: spectral width of 6578 Hz, 512 *t*_1_ increments with 24 transients of 4096 points for LyeTx I-b_cys_ and spectral width of 6849 Hz, 512 *t*_1_ increments with 40 transients of 4096 points for LyeTx I-bPEG.

^1^H-^13^C Heteronuclear Single Quantum Coherence (^1^H-^13^C HSQC) spectral data were acquired in phase-sensitive fashion such that CH and CH_3_ correlation show positive and CH_2_, negative (Willker et al., 1993). Regarding the acquisition parameters for LyeTx I-b_cys_ sample, F1 and F2 spectral widths of 15,822 and 6578 Hz were used, respectively; 512 *t*_1_ increments with 24 transients of 2048 points. In the case of the LyeTx I-bPEG samples, F1 and F2 spectral widths of 15,822 and 6849 Hz, 416 *t*_1_ increments with 24 transients of 2048 points were used. ^1^H-^15^N Heteronuclear Single Quantum Coherence (^1^H-^15^N HSQC) data (Fairbrother et al., 1991; Schleucher et al., 1994) were acquired using F1 and F2 spectral widths of 2311 and 6578 Hz, respectively, 88 *t*_1_ increments with 400 transients of 1024 points for LyeTx I-b_cys_ and using F1 and F2 spectral widths of 2554 and 6849 Hz, respectively, 80 *t*_1_ increments with 464 transients of 1024 points for LyeTx I-bPEG.

Data acquisition was performed using Bruker Topspin, and data processing was performed using NMRPipe 10.9 (Delaglio et al., 1995).

Assignments of the ^1^H, ^13^C, and ^15^N resonances were performed by simultaneously analyzing the respective set of NMR contour maps of each peptide using NMRViewJ (Johnson and Blevins, 1994). The intensities of the NOE correlations of the NOESY spectra of LyeTx I-b_cys_ and LyeTx I-bPEG, recorded at mixing times of 150 ms, were converted to semi-quantitative distance restraints according to Hyberts et al. (1992). Upper limits of 2.8, 3.4 and 5.0 Å restraints correspond to strong, medium and weak NOEs, respectively. Additional geometric restraints were determined from the chemical shifts of C_ɑ_, H_ɑ_, C_β_, N, and HN using TALOS+ (Shen et al., 2009). The three-dimensional structures of LyeTx I-b_cys_ and LyeTx I-bPEG were calculated using a simulated annealing protocol available as part of Xplor-NIH-version 2.17 (Schwieters et al., 2003, 2006). The calculations began with an extended conformation subjected to 19,000 steps of simulated annealing at 1,000 K followed by 11,000 steps of temperature reduction in the first slow cooling (annealing) phase. The ten geometries with the lowest energy were selected from the group of two hundred calculated structures, and the stereochemical quality of each of these geometries was checked using PROCHECK-NMR (Laskowski et al., 1996). The graphical representation of the obtained structural ensemble was made using MOLMOL (Koradi et al., 1996) and PyMOL (Schrödinger, 2015).

### 2.13 Experiments for the degradation assay

The enzymes used in the experiments were trypsin and proteinase K. Enzyme solutions were prepared a few minutes before the experiments and contained 1.0 µM enzyme in 10 mM phosphate buffer at pH 8.0 for activation. Comparative proteolysis experiments between LyeTx I-b and LyeTx I-bPEG were performed by digesting the peptide solution (50 μM) with the enzyme solutions incubated at 37°C at an enzyme:substrate ratio of 1:50 (w/w) for trypsin and 1:50 (w/w) for proteinase K for 24 h. Inactivation of enzymes was achieved at pH 2.0—3.0 (Smillie and Neurath, 1959) by addition of 1.0 M hydrochloric acid solution. After inactivation, aliquots of 50 µL were injected into a Varian™ chromatograph (Varian, Inc. Corporate Headquarters, Palo Alto, CA, United States), model Pro Star 315, with ultraviolet detector, model Pro Star 335, using a Vydac™ 218 TP C18 (250 × 4.6 mm) reverse phase column. Chromatographic analyses were performed using a gradient of deionized water containing 0.1% TFA and acetonitrile containing 0.08% TFA. Samples were detected at 215 nm under a flow of 0.80 mL · min^−1^ for 45 min.

## 3 Results

### 3.1 Synthesis and purification of LyeTx I-b and LyeTx I-b_cys_

To prepare LyeTx I-b for targeted conjugation with polyethylene glycol, Leu-21 was replaced with a cysteine residue so that the PEGylation reaction could occur at this specific site. The obtained gross yield was about 50% (100 mg). The cysteine-containing peptide (LyeTx I-b_cys_) was purified by RP-HPLC and was eluted at a retention time of 26.61 min. After purification (Supplementary Figure S1A), the peptides were collected, analyzed by MALDI-ToF-MS (Supplementary Figures S1B,C), and lyophilized. Confirmation of the amino acid sequence of LyeTx I-b_cys_ was obtained by MS/MS. After purification by RP-HPLC, the product was lyophilized with final a yield about 65%.

The subsequent PEGylation of LyeTx I-b_cys_ was performed using 2 kDa PEG-containing mPEG-MAL specifically since this molar mass was deemed not too small as to not affect biological properties and not too large, which could shield the peptide pharmacophore from its intended target.

### 3.2 Activity of LyeTx I-b_cys_ and LyeTx I-bPEG against gram-positive and -negative bacteria

To further test whether the substitution of Leu-21 for a cysteine residue and the corresponding PEGylation (Figure 1) affects antimicrobial activity, the minimum inhibitory concentrations (MIC) for both peptides were evaluated. The assays yielded identical MIC values for LyeTx I-b_cys_ and LyeTx I-b peptides, 1.0 μM for *E. coli* ATCC 25922 and 2.0 μM for *S. aureus* ATCC 33591 (Table 1). LyeTx I-bPEG showed a fourfold reduced activity against *E. coli* ATCC 25922 and *S. aureus* ATCC 33591 compared to LyeTx I-b_cys_ (Table 1). The decrease of activity was also observed for *E. coli* and *S. aureus* clinical isolates. Additionally, previous studies have shown that peptide PEGylation was fortunately able to maintain activity against carbapenem-resistant *Acinetobacter baumannii* while reducing cytotoxicity (Brito et al., 2021).

**TABLE 1:** Minimum inhibitory concentration (MIC), cytotoxic concentration (CC_50_) and selectivity index (SI) of LyeTx I-b, LyeTx I-bPEG and MIC of LyeTx I-b_cys_, in µM.

	LyeTx I-b			LyeTx I-b_cys_	LyeTx I-bPEG		
	MIC	CC_50_	SI	MIC	MIC	CC_50_	SI
*E.coli* ATCC 25922	1.0	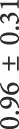	0.96	1.0	4.0	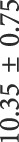	2.59
*E.coli* ISOL_10	1.0		0.92	1.0	4.0		2.59
*E.coli* ISOL_11	1.0		0.96	1.0	4.0		2.59
*S. aureus* ATCC 33591	2.0		0.48	2.0	8.0		1.29
*S. aureus* ISOL_1	2.0		0.48	2.0	8.0		1.29

### 3.3 Evaluation of cellular viability using MTT technique for VERO cells

The PEGylated peptide exhibited a more than 10-fold increase in CC_50_ values for VERO cells, compared with LyeTx I-b_cys_, which represents a significant reduction in cytotoxic activity for monkey renal cells (Table 1). This reduction in cytotoxicity is noteworthy because drugs must have low toxicity to mammalian cells of a variety of organs, including renal cells, which were used as an *in vitro* model in this work.

### 3.4 Effect of PEGylation on peptide-membrane interactions

**FIGURE 2:**
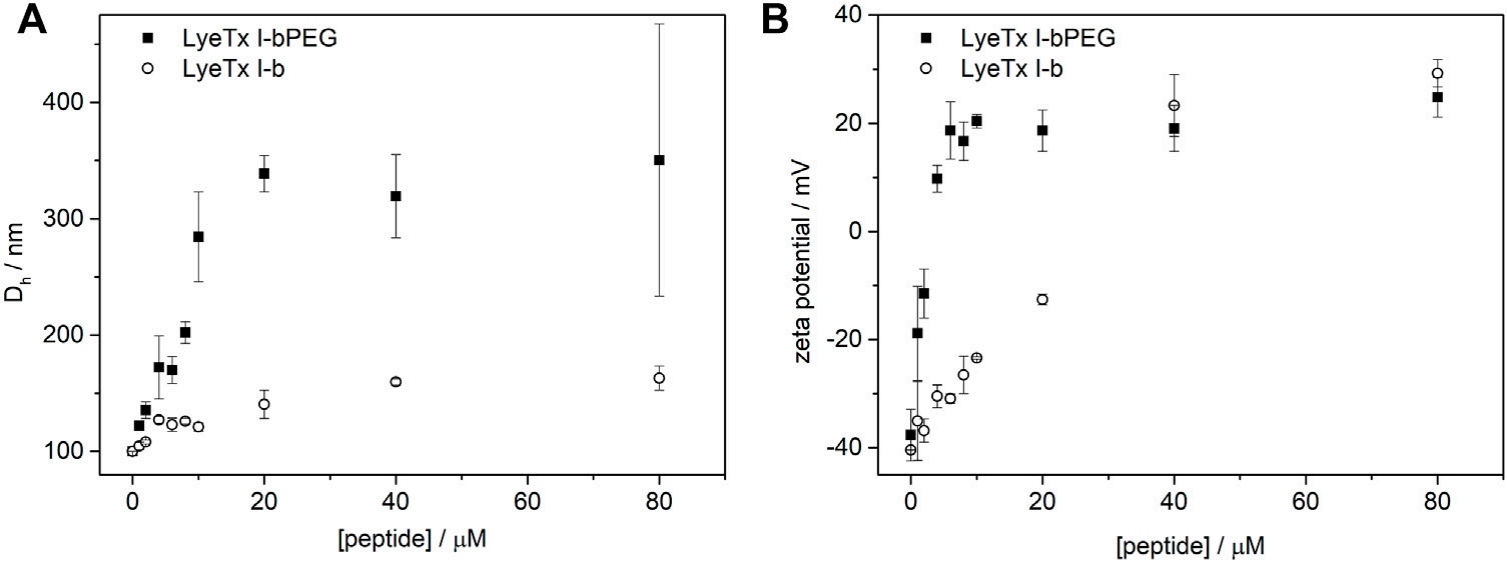
**(A)** Hydrodynamic diameter (*D*_h_) and **(B)** normalized zeta potential (*ζ*-potential) of POPC:POPG LUVs as a function of (circles) LyeTx I-b or (squares) LyeTx I-bPEG concentration. LUVs containing 500 µM were suspended in 10 mM Tris-HCl buffer pH 8.0 at 25°C. Error bars represent the standard deviation of three independent experiments.

The effect exerted by each peptide on the anionic membranes was investigated by DLS and ζ-potential measurements, assessing the hydrodynamic diameter (*D*_h_) and neutralization of 500 µM POPC:POPG (3:1) LUVs. We chose the conditions for studying peptide-membrane interactions by DLS experiments considering the range encompassing the lowest and highest MIC values obtained for the PEGylated form in biological tests against *E. coli* (1.0 µM) and *S. aureus* (8.0 µM) as well as higher peptide concentrations in bacterial strains. Figure 2 shows the effect of peptides on the *D*_h_ and ζ-potential of POPC:POPG LUVs, with all data showing a polydispersity index (PDI) below 0.3, indicating a homogeneous size distribution. The interaction of LyeTx I-b or LyeTx I-bPEG with LUVs leads to an increase in both the *D*_h_ (Figure 2A) and ζ-potential (Figure 2B) of phospholipid vesicles (Manzini et al., 2014). Notably, LyeTx I-bPEG causes a considerably greater increase in hydrodynamic diameter (Δ*D*_h_ ≅ 200 nm) compared to LyeTx I-b (Δ*D*_h_ <50 nm). As expected, the addition of the cationic peptides increased the membrane surface charge of the anionic POPC:POPG LUVs even at low peptide concentrations. Again, LyeTx I-bPEG has a stronger effect on the ζ-potential compared to LyeTx I-b. Consequently, neutrality of LUVs is achieved upon addition of 2–4 μM of the PEGylated form, whereas a similar effect for LyeTx I-b is observed only at a concentration range of 20–40 μM.

Calcein leakage studies were conducted for LyeTx I-b and LyeTx I-bPEG in the presence of POPC and POPC:POPG 3:1 LUVs (Supplementary Figure S2). It is immediately observable that LyeTx I-bPEG promotes less calcein release than LyeTx I-b either in the presence of POPC:POPG 3:1 (Supplementary Figure S2A) or POPC (Supplementary Figure S2B) LUVs. Specifically, the PEGylated derivative presented an 8% leakage in the presence of the zwitterionic medium and 68% in the anionic, while LyeTx I-b, 15 and 94%, respectively.

The aspects of peptide-membrane interactions of LyeTx I-b and the PEGylated form with anionic phospholipid LUVs was investigated by SPR. POPC:POPG (3:1) LUVs were interacted with an SPR gold sensor chip covered with a dithiothreitol (DTT) monolayer. LUVs were immobilized on *ex situ*-prepared DTT-gold chip sensors with 50 μL · min^−1^ buffer flow (10 mM Tris–HCl, pH 8.0 running buffer) at 25°C. Pure phospholipid LUVs gave higher values of *RU* signals, which stabilized after approximately 15 min of injection.

**FIGURE 3:**
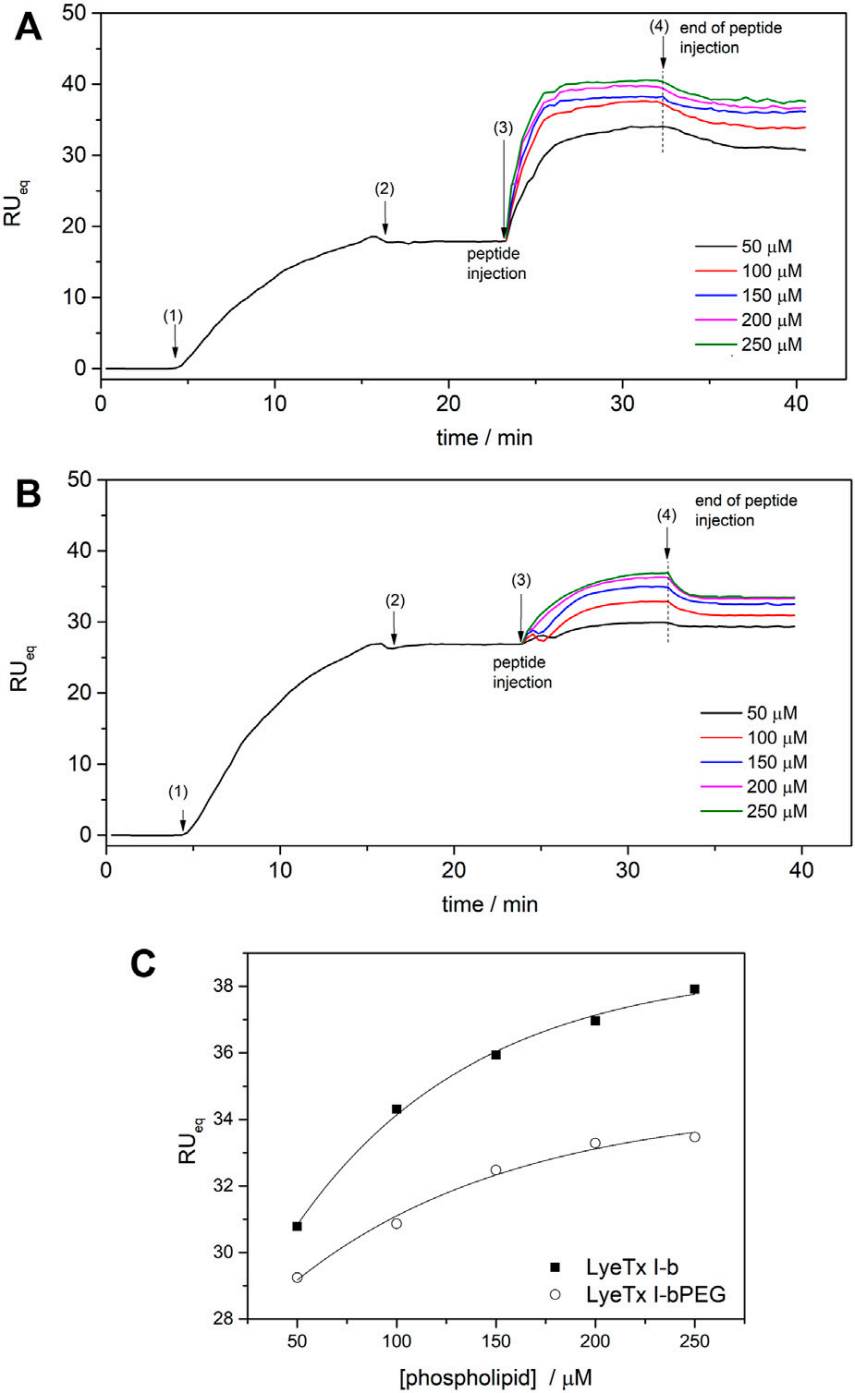
SPR sensograms for the bilayer interaction of **(A)** LyeTx I-b and **(B)** LyeTx I-bPEG. POPC:POPG LUVs (0.05—0.25 mM) were immobilized on the surface of the DTT-gold sensor chip and 10 µM peptide solutions were injected at a flow rate of 10 ml min^−1^. **(C)** The dependence of *RU*_eq_ intensities of (square) LyeTx I-b and (circle) LyeTx I-bPEG on lipid concentration. The numbers correspond to (1) start of LUV injection, (2) end of LUV injection, (3) start of peptide injection, and (4) end of peptide injection. Solid lines are simulations according to Eq. 2, accounting for surface-peptide electrostatics: *K*_LyeTx I-b_ = 6800 ± 500 M^−1^, *K*_LyeTx I-bPEG_ = 3500 ± 500 M^−1^.

Injections of both peptides onto phospholipid surfaces resulted in an increase in *RU* signal intensity as a function of phospholipid concentration, confirming peptide-membrane interactions (Junior et al., 2017). Although the profiles of the peptide-membrane binding sensograms of both peptides are similar, significantly higher response values are observed for the membrane interactions of LyeTx I-b (Figure 3A) compared to LyeTx I-bPEG (Figure 3B).

Surface partition coefficients (*K*) of the interactions between peptides and anionic model membranes were determined by simulations according to Eq. 2, where *RU* signal intensity was considered as a function of phospholipid concentration (Figure 3). The SPR titrations resulted in *K* of 6800 M^−1^ and 3500 M^−1^ for LyeTx I-b and LyeTx I-bPEG, respectively. The interaction between the peptides and POPC LUVs was too weak to be observed by SPR experiments and, therefore, *K* values were not obtained in this case.

### 3.5 Structural analysis of LyeTx I-b_cys_ and LyeTx I-bPEG by NMR in solution

Solutions of LyeTx I-b_cys_ and LyeTx I-bPEG in TFE-*d*_2_:H_2_O 60:40 were prepared to obtain the respective NMR spectra. Aqueous TFE solutions are known to induce the formation of α-helix structures. Sequence-specific chemical shift assignments were performed for the free and PEGylated using simultaneous analysis of TOCSY and NOESY spectra (Wüthrich, 1986). Concomitantly, the heteronuclear spectra were used as extra control to characterize unequivocally the spin systems of each amino acid residue. For instance, the inspection of ^1^H-^13^C HSQC spectra allows the clear separation of C_*α*_ H_*α*_ correlations related to isoleucine and leucine residues, as they show, respectively, ^13^C resonances at 61.6 and 55.7 ppm in the respective edited spectrum (Supplementary Figure S3). In its turn, the respective spin systems are promptly recognized in the respective homonuclear spectra (Supplementary Figure S3).

**FIGURE 4:**
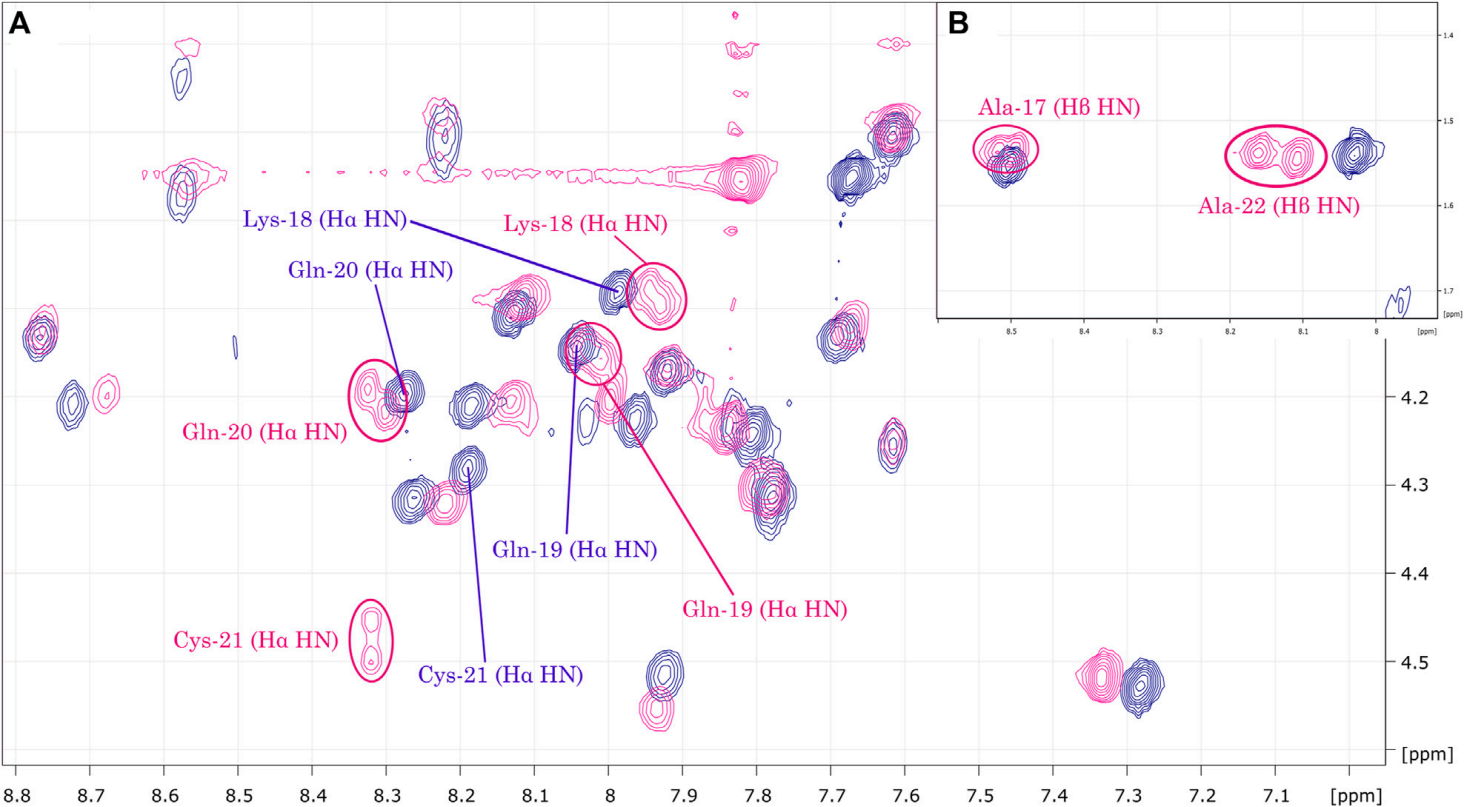
**(A)** Overlay of LyeTx I-b_cys_ (dark blue) and LyeTx I-bPEG (pink) TOCSY contour maps. The highlighted correlations **(B)** range from Ala-17 to Ala-22 and show that mPEG-MAL conjugation resulted in signal broadening or splitting for Hα.HN and, for alanine residues, Hβ.HN correlations in LyeTx I-bPEG, while this was not observed for LyeTx I-b_cys_.

The obtained NMR spectra are characterized by a high number of sufficiently distributed correlations, suggesting well-folded conformations. Analysis of the TOCSY contour maps (Figure 4) reveals Hα.HN correlations for residues Ala-17 to Ala-22 of LyeTx I-bPEG that are broadened or explicitly duplicated. The same pattern of differences in chemical shift, signal broadening and duplication was observed in the ^1^H-^15^N HSQC contour maps (Supplementary Figure S4) for both peptides.

**FIGURE 5:**
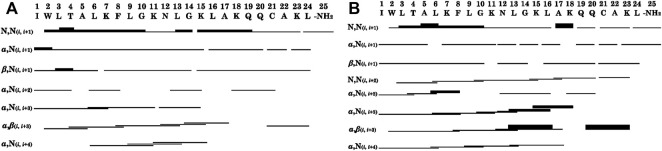
Graphical summary of NOE correlations characteristic of α-helical structures observed in NOESY contour maps of **(A)** LyeTx I-b_cys_ and **(B)** LyeTx I-bPEG. Thin, medium and thick bars represent weak, medium and strong NOE correlations, respectively. NH_2_ represents the carboxamide terminus.

The summaries of the inter-residue NOE correlations of LyeTx I-b_cys_ and LyeTx I-bPEG (Figure 5) are characteristic of α-helical secondary structures. Analysis of the helical percentages for each residue based on the experimental chemical shift values was performed using TALOS+ (Shen et al., 2009), which yielded an α-helical segment extending from Trp-2 to Lys-23 for both peptide chains (Supplementary Figure S5).

**FIGURE 6:**
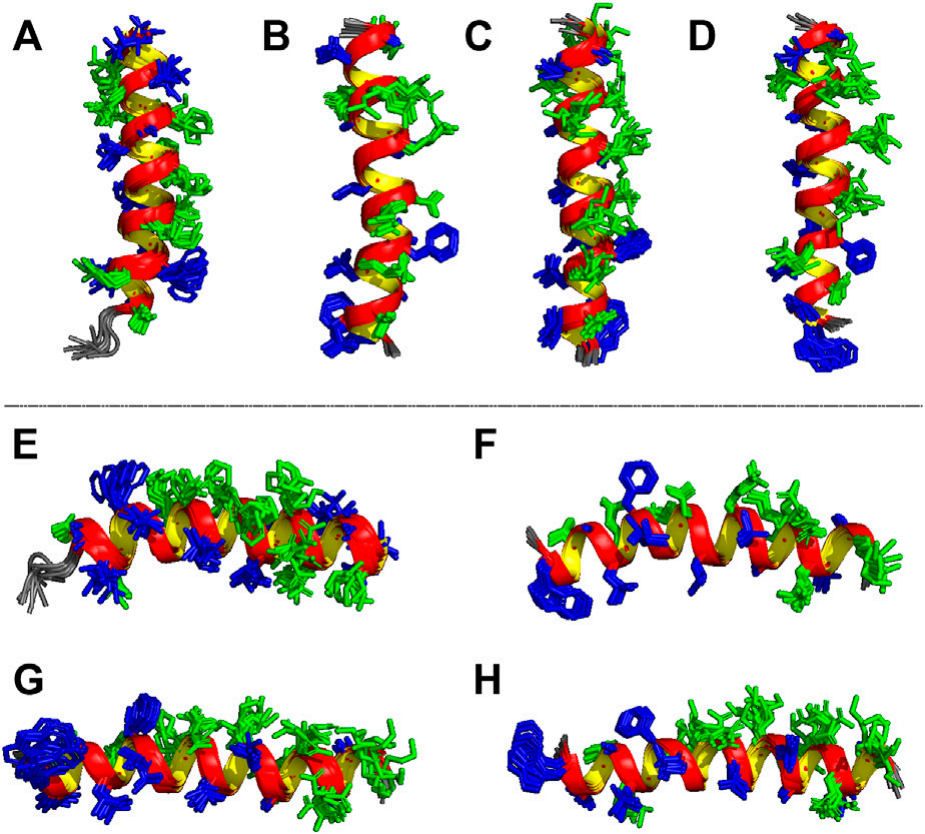
Ensembles of the 10 most stable structures of **(A,E)** LyeTx I (Santos et al., 2010, PDB ID 7MMM), **(B,F)** LyeTx I-b (Reis et al., 2018, PDB ID 6CL3), **(C,G)** LyeTx I-b_cys_ and **(D,H)** LyeTx I-bPEG. Panels **(A–D)** show the vertical perspective of each helical structure with each respective *N*-terminus facing down while panels **(E–H)** represent the horizontal view with each *N*-terminus to the left and *C*-terminus to right. Hydrophobic residues are shown in dark blue and hydrophilic residues in green.

Based on the distance and dihedral angle restraints derived from the NOE and chemical shift data, the three-dimensional structures of LyeTx I-b_cys_ and the PEGylated peptide were obtained using a simulated annealing protocol (Figures 6C,D,G,H), and the results are compared with the NMR structures of LyeTx I-b and the wild-type peptide (Figures 6A,B,E,F). Both calculated structures are characterized by a well-defined helical segment covering most of the polypeptide sequence, and the obtained structural ensembles for LyeTx I-b_cys_ and LyeTx I-bPEG showed relatively low RMSD values for the superposition of all heavy atoms or backbone atoms only (Table 2). All *φ/ψ* dihedral angle pairs are in the most favorable regions of the Ramachandran plot (Supplementary Figure S6), indicating the high stereochemical quality of the calculated structures, and the summary of their structural statistics is shown in Table 2.

**TABLE 2:** Summary of structural statistics of LyeTx I-b_cys_ at 2.0 mM and LyeTx I-bPEG at 1.5 mM in TFE-*d*_2_:H_2_O (60:40) at 20°C, pH 7.0 (phosphate buffer at 20.0 mM).

		
	**LyeTx I-b_cys_**	**LyeTx I-bPEG**
Total number of restraints	490	530
Number of intraresidue restraints	323	330
Number of sequential restraints (*i*, *i* + 1)	123	124
Number of medium range restraints (*i*, *i* + *j*)_*j* = 2, 3, 4_	44	76
RMSD (Å)—all residues		
Backbone	0.85 ± 0.25	0.71 ± 0.22
Backbone and heavy atoms	1.62 ± 0.30	1.45 ± 0.25
RMSD (Å)—helical segment		
Backbone	0.72 ± 0.22	0.59 ± 0.19
Backbone and heavy atoms	1.49 ± 0.28	1.29 ± 0.24
Ramachandran plot analysis		
Residues in most favored regions	100%	100%
Residues in additional allowed regions	0	0
Residues in generously allowed regions	0	0
Residues in disallowed regions	0	0

**FIGURE 7:**
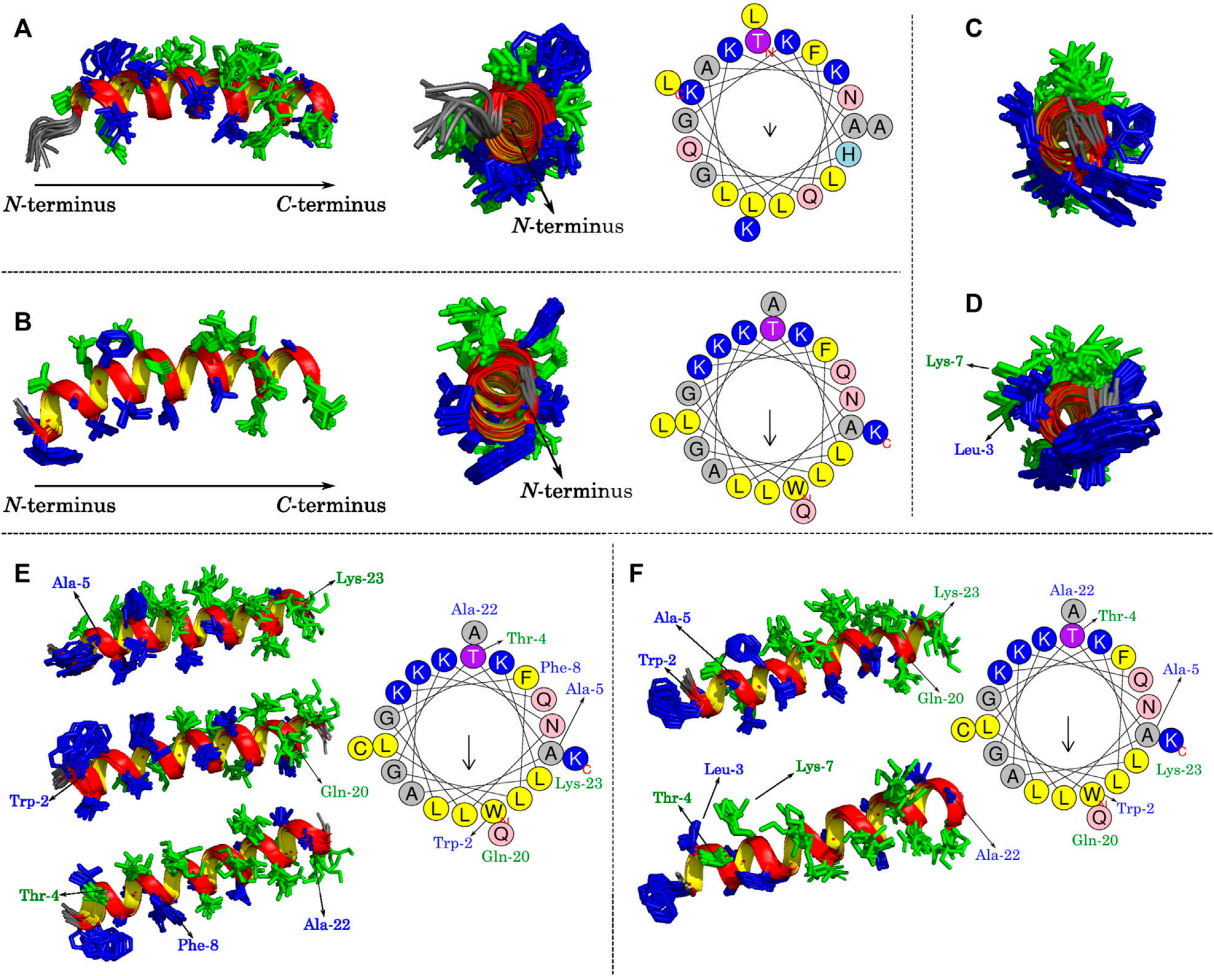
Lateral and frontal views of **(A)** LyeTx I (Santos et al., 2010) and **(B)** LyeTx I-b (Reis et al., 2018) helix structures with their respective Schiffer-Edmundson projections from HelixQuest. Frontal views of **(C)** LyeTx I-b_cys_ and **(D)** LyeTx I-bPEG helices are shown and panels **(E)** and **(F)** explicitly show the residues involved in disrupting the hydrophobic and hydrophilic faces of LyeTx I-b_cys_ and LyeTx I-bPEG, respectively, as verified by their respective Schiffer-Edmundson projections. In all helix plots, the hydrophobic residues are shown in dark blue and the hydrophilic ones in green.

Some structural features of LyeTx I, the wild-type peptide present in the toxin of *Lycosa erythrognata*, its derivative LyeTx I-b, LyeTx I-b_cys_, and LyeTx I-bPEG can be noticed by analyzing Figures 7A–F. LyeTx I (Santos et al., 2010, PDB ID 7MMM) does not exhibit a significant amphipathic character, as evidenced by the absence of a long helix segment composed solely of either hydrophobic or hydrophilic residues (Figure 7A), which, in this case, appear to be stochastically distributed. LyeTx I-b, on the other hand, shows a clearer partitioning between a hydrophobic and a hydrophilic surface, although their continuity is interrupted by Gln-20 and Ala-22, respectively (Figure 7B). In comparison to LyeTx I, LyeTx I-b_cys_ showed no significant change in amphipathicity, which was to be expected since the only chemical modification was the replacement of Leu-23 with a cysteine residue (Figures 7C,E). However, the conjugation with the PEG group introduced some structural features, such as the fact that the Leu-3 and Lys-7 residues, which were far apart and had no significant overlap in LyeTx I-b_cys_, are almost eclipsed in the PEGylated structure (Figure 7D), breaking the continuity of the hydrophilic face. Besides, the Michael addition shifted the position of some residues away from their calculated Schiffer-Edmundson projection positions, such as Trp-2 and Ala-5, which overlap with Lys-23 in the structure of LyeTx I-bPEG **(** Figure 7D).

### 3.6 Degradation assays by peptidases

**FIGURE 8:**
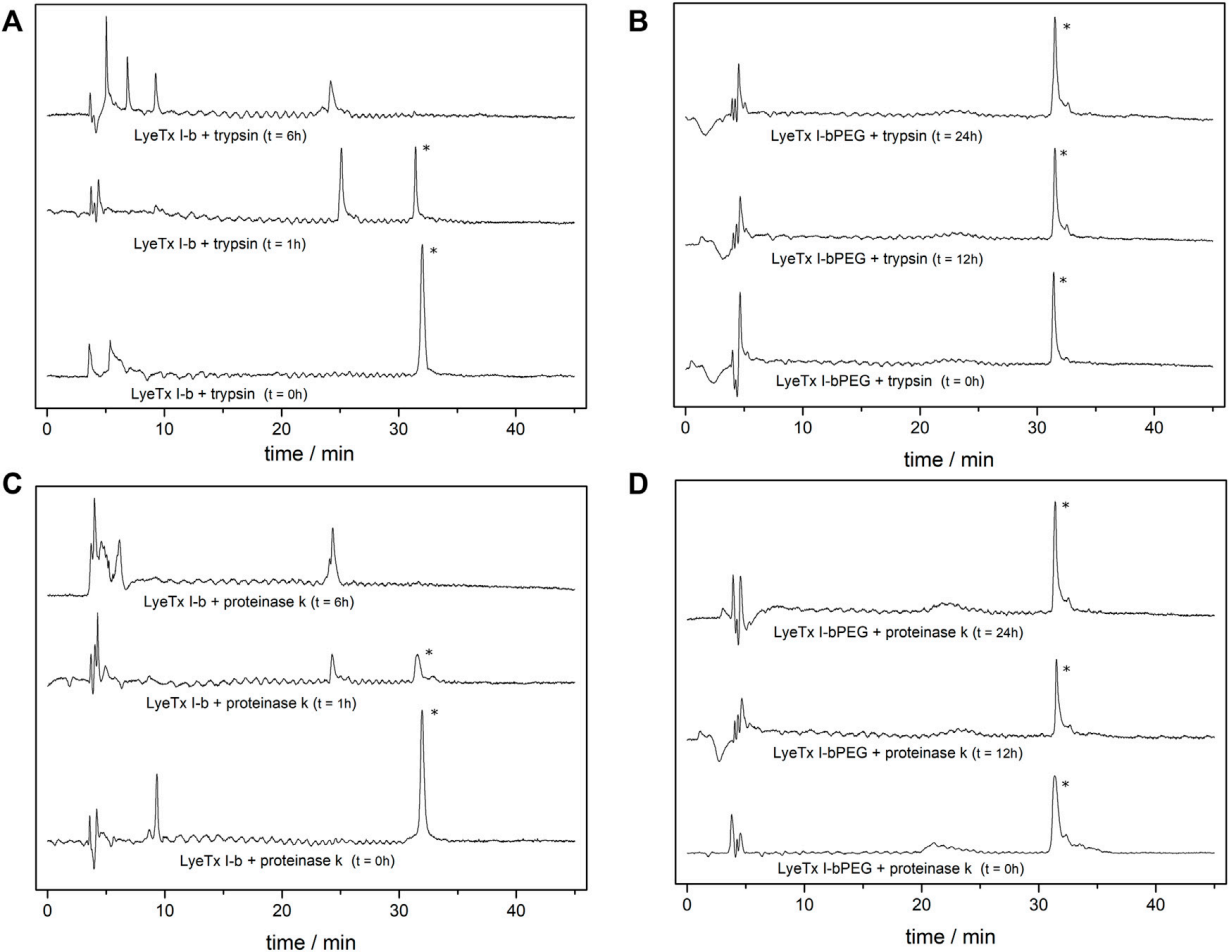
Comparative proteolytic degradation of LyeTx I-b and LyeTx I-bPEG. Peptides at 50 mM concentrations were incubated for 24 h with 1:50 (enzyme to substrate) weight ratio of **(A)** trypsin, **(C)** proteinase K, and 1:25 (enzyme to substrate) weight ratio of **(B)** trypsin and **(D)** proteinase K, all in 10 mM potassium phosphate, pH 8.0, at 37°C.

Proteolysis experiments were performed to evaluate the effects of PEGylation on the degradation of LyeTx I-bPEG. For this purpose, LyeTx I-b and LyeTx I-bPEG were exposed to trypsin and proteinase K enzymes for 24 h at 37°C. In time-course proteolysis experiments, both peptides were digested at 50 µM with an enzyme-to-substrate ratio of 1:50 (w/w) for trypsin and 1:25 (w/w) for proteinase K (Figure 8). The enzyme kinetics of trypsin and proteinase K are similar for both peptides. While LyeTx I-bPEG could not be digested in the presence of either trypsin or proteinase K, LyeTx I-b was almost completely degraded after 6 h of incubation with either trypsin or proteinase K.

## 4 Discussion

The search for broad-spectrum antimicrobial agents is a public health priority worldwide due to the flood of multidrug-resistant bacteria, and with this in mind, a variety of matrices are undergoing clinical trials to identify their antimicrobial potential (Burki, 2018). Nevertheless, many molecules exhibit marked broad-spectrum antimicrobial and cytotoxic activity, as is the case with LyeTx I-b, which may negate their use *via* parenteral routes. Previous studies have shown that peptide PEGylation was fortunately able to maintain activity against carbapenem-resistant *Acinetobacter baumannii* while reducing cytotoxicity (Brito et al., 2021). The present work goes a step further and demonstrates the antimicrobial activity of the PEGylated peptide against Gram-positive and Gram-negative bacteria, as well as the reduction of toxicity against VERO cells. In addition, a more robust chemical, biophysical and structural characterization of LyeTx I-bPEG is presented.

LyeTx I-b_cys_ yielded MIC values similar to those obtained for LyeTx I-b by Reis et al. (2018) and smaller than those for LyeTx I reported by Santos et al. (2010); Fuscaldi et al. (2016). These results show that antimicrobial activity was maintained even though Leu-21 was replaced by a cysteine residue. MIC values obtained for both synthesized peptides against *S. aureus* and *E. coli* were similar to those obtained for LyeTx I (Santos et al., 2010; Fuscaldi et al., 2016) and LyeTx I-b (Reis et al., 2018). Thus, modification of Leu-21 to a cysteine preserved antimicrobial activity. This was expected, since Fuscaldi et al. (2016) demonstrated that a modification near the *C*-terminus, coupling a HYNIC group, did not affect the antimicrobial properties of LyeTx I.

The decrease of antimicrobial activity in the PEGylated peptide was also expected, as previously reported by Kodera (1998). According to these authors, PEGylation of the antimicrobial peptide lysostaphin preserved 47% of its original activity. However, the loss of *in vitro* activity is compensated by the lower toxicities *in vitro* and *in vivo* (Brito et al., 2021).

Biophysical and NMR studies of LyeTx I-b and LyeTx I-bPEG were performed to evaluate the effects of PEGylation on the peptide structure and peptide-membrane interactions. The biophysical results regarding the membrane interactions of LyeTx I-b and LyeTx I-bPEG showed that PEGylation resulted in a decrease in affinity for negatively charged membranes. The lower affinity coefficient (*K*) observed in the SPR experiments for LyeTx I-bPEG (3500 M^−1^) compared with LyeTx I-b (6800 M^−1^) suggests that the PEG chain may hinder the interaction with the phospholipid bilayer.

Peptide-membrane interactions can lead to changes in the organization and net charge of the phospholipid membrane surface, as reflected by a change in the *D*_h_ and *ζ*-potential of phospholipid vesicles (Freire et al., 2011; Munhoz et al., 2021). Therefore, affinity coefficients determined by SPR correlate directly with the *D*_h_ and *ζ*-potential of POPC:POPG LUVs, with both values changing after addition of LyeTx I-b and LyeTx I-bPEG. Although the PEGylated form exhibited higher changes in the *ζ*-potential of LUVs at lower concentrations (<40 mM), both peptides resulted in a similar increase (Δ*ζ* ≈ 60 mV) at higher concentrations (>40 mM). These results suggest that LyeTx I-b and LyeTx I-bPEG exhibit a similar mode of interaction with the membrane, which is consistent with the NMR data, as no significant differences are observed when comparing the respective three-dimensional structures. Interestingly, higher *D*_h_ variations were observed for POPC:POPG LUVs after addition of LyeTx I-bPEG for all tested concentrations. Since a lower affinity coefficient and *in vitro* activity were observed for LyeTx I-bPEG, the PEG moiety might be uninvolved from the point of view of interaction with the membrane surface and, consequently, contributes to the *D*_h_ increase of the LUVs (Immordino et al., 2006). The high polydispersity index PDI values (>0.3) and an additional size pattern around 8 µm (Supplementary Figure S7) at high LyeTx I-bPEG concentrations (above 60 µM) indicate the occurrence of an aggregation phenomenon, which is responsible for the high *D*_h_ values observed.

Additionally, considering the results obtained in calcein release assays (Supplementary Figure S2), it can be inferred that vesicle fusion does not take place, since smaller fluorescence intensities were observed for LyeTx I-bPEG in the presence of POPC and POPC:POPG LUVs. On the other hand, the higher calcein release values for LyeTx I-b (15% and 94% for POPC and POPC:POPG LUVs, respectively) suggest greater lytic activity values for this peptide when compared to LyeTx I-bPEG, in accordance with the smaller MIC. Furthermore, the higher fluorescence intensity observed for both peptides in the presence of the negative biomimetic medium reveals that electrostatic interactions play an important role in the membrane interactions of these peptides.

As described in a recent publication (Brito et al., 2021), conjugation of the mPEG-MAL moiety anchored by the maleimidyl group to the thiol group of cysteine-21 of LyeTx I-b_cys_ was confirmed by NMR, MALDI-ToF, and RP-HPLC analyses. The Nuclear Magnetic Resonance spectra of both the precursor LyeTx I-b_cys_ and LyeTx I-bPEG showed significant changes in the spectral profile and chemical shifts, especially of ^1^H nuclei, confirming the completion of the reaction. Clear evidence was the absence of the olefinic signals in the ^1^H NMR spectrum of the PEGylated derivative and the presence of succinimidyl-related signals in its corresponding H-^13^C HSQC spectrum. In addition, the broadening or doubling of some resonances related to Cys-21 and oer residues near the peptide-PEG linkage, such as Gln-20 and Ala-22, were observed. These results were attributed to the formation of epimers, which were expected according to the stereochemical aspects of Michael addition as a new chiral center is formed (Nair et al., 2014).

Only intra-residue and sequential (*i*, *i* + 1) correlations were assigned in our previous NMR analysis (Brito et al., 2021), which focused on the characterization of spin system to confirm the peptide-PEG conjugation. In the present work, we analyzed the NMR spectra in more detail to obtain information about the three-dimensional structure of LyeTx I-b_cys_ and LyeTx I-bPEG. Their spectra showed a high number of sufficiently dispersed correlations, indicating well-folded conformations for both. In this context, we assigned medium-range correlations present in the respective NOESY spectra.

When superimposing the TOCSY contour maps of LyeTx I-b_cys_ and LyeTx I-bPEG, the Hα.HN correlations for residues Ala-17 to Ala-22 of the PEGylated form are either broadened or duplicated, as they are for Gln-20, Cys-21, and Ala-22, confirming that mPEG-MAL conjugation led to epimers. Comparing the respective ^1^H-^15^N HSQC spectra, the differences in chemical shifts and the broadening or doubling of correlations involving nuclei of this peptide segment also confirm the conjugation of LyeTx I-b_cys_ with mPEG-MAL and the formation of epimers.

In both cases, many (*i*, *i* + 1) correlations are observed, spanning almost the entire peptide sequence. In addition, a large number of medium-range NOE interactions was detected, including (*i*, *i* + 3) and (*i*, *i* + 4) correlations extending from the acylated *N*-terminus to Ala-17. Assignment of some medium-range NOE correlations extending from Ala-17 to the *C*-terminus of the peptide was complicated by a strong overlap of chemical shifts in the corresponding region of the NOESY contour map. Nevertheless, an α, β (*i*, *i* + 3) correlation involving Gln-20 and Lys-23 strongly suggests the existence of a structural segment in both the free and mPEG-conjugated peptide near Cys-21.

To obtain further structural information, an analysis of secondary structure preferences based on chemical shift data was performed using TALOS+ (Shen et al., 2009), which revealed, with high confidence, an α-helical segment extending from Trp-2 to Lys-23 (Supplementary Figure S5). Similar to LyeTx I-b (Reis et al., 2018), acylation at the *N*-terminus and the carboxamide moiety at the *C*-terminus appear to provide structural stability at the LyeTx I-bPEG termini.

Interestingly, mPEG-MAL conjugation did not result in a significant degree of conformational freedom of the polypeptide chain, which retains its overall structural properties. This is a positive feature since the biological activity of AMPs largely depends on their three-dimensional structures. Besides, the high structural similarities are in line with the conservation of the PEGylated species affinity of anionic membranes, as indicated by the DLS, SPR, and *ζ*-potential results.

The high helicity observed for LyeTx I-b_cys_ and LyeTx I-bPEG was previously observed in LyeTx I-b (Reis et al., 2018). In this context, the structural effect of acylation at the *N*-terminus is clear, as it stabilizes the positive end of the helix dipole by eliminating its net positive charge. Moreover, the carbonyl group of the resulting acetamide interacts with the amide hydrogens near the *N*-terminus *via* hydrogen bonds that locally stabilize the helical segment. Interestingly, a slightly curved helix can be seen in the structure of LyeTx I-bPEG, which is due to some HN.HN (*i*, *i* + 2) correlations observed in the NOESY spectrum of the PEGylated peptide but absent in the corresponding spectrum of LyeTx I-b_cys_. An even higher number of this type of correlation was observed for LyeTx I-b (Reis et al., 2018).

While the chemical modifications of LyeTx I to LyeTx I-b and LyeTx I-b_cys_ increased the overall amphipathic character of the biopolymer, the PEGylation process resulted in some decrease. However, direct correlations between high amphipathicity and biological activity are not readily established for AMPs, since ocellatins (Gomes et al., 2018) are examples which are not highly amphipathic in nature but show pronounced membrane affinity. The activity of the PEGylated peptide can therefore be understood as the sum of different structural and chemical aspects such as: amphipathicity, net charge, presence or absence of charged residues, helical content, and the presence of chemically active moieties such as PEG. All these can act on microorganisms with mechanisms of action different from usual AMPs or improve structural resilience to different forms of degradation.

LyeTx I shows a high degree of structural flexibility, as its ensemble is not as ordered as that of LyeTx I-b and the peptide moiety of LyeTx I-bPEG. In addition, although not as flexible as LyeTx I, LyeTx I-b_cys_ also reveals some relative conformational freedom. These structural observations are supported by the RMSD values for each structural ensemble of the four peptides considering all residues and only the helical segments (Supplementary Table S2). Accordingly, LyeTx I is the most flexible of the four and LyeTx I-b the least flexible, whereas, interestingly, the Leu/Cys modification significantly increases flexibility and PEGylation promotes better structural cohesion within the peptide moiety.

The helical amphipathic secondary structure of LyeTx I-bPEG suggests that the interaction between the peptide and the membrane likely occurs when its main helical axis and the membrane surface are parallel. Therefore, the introduction of a considerably large group like PEG can sterically block the interacting face of the peptide. This is in line with the obtained affinity coefficients (*K*) of the SPR experiments for LyeTx I-b (6800 M^−1^) and LyeTx I-bPEG (3500 M^−1^) in the presence of anionic LUVs, revealing a smaller affinity for the PEGylated derivative. Likewise, the same PEG-induced spatial impairment can be the cause of a smaller lytic activity for LyeTx I-bPEG, as evidenced by its smaller calcein release percentage. Nevertheless, the interaction mechanism of LyeTx I-b, mainly driven by electrostatic forces (Reis et al., 2021), does not seem to be significantly altered after PEGylation, since a smaller calcein release was observed for both peptides in the presence of zwitterionic LUVs in comparison with anionic LUVs.

Finally, the enzymatic degradation experiments showed improved proteolytic resistance acquired after PEGylation, as LyeTx I-bPEG showed great stability in the presence of trypsin or proteinase K even after 24 h of exposure, whereas LyeTx I-b was completely degraded after 6 h in the presence of either enzyme. Thus, although a partial loss of antimicrobial activity was observed upon PEGylation, mainly due to the decrease in membrane affinity, the PEG moiety did not significantly alter the nature of the peptide-membrane interaction, while still conferring impressive proteolytic resistance to the peptide chain.

## 5 Conclusion

Substitution of Leu-21 for a cysteine residue in the APM LyeTx I-b followed by PEGylation using mPEG-MAL gave an overall satisfactory result, and multiple PEGylations were not detected. The structural features of LyeTx I-bPEG, such as the long helical segment and the distribution of residues, were largely preserved compared with LyeTx I-b, although slightly lower amphipathicity was observed for the PEGylated derivative. In addition, a small degree of structural freedom was introduced by the substitution of Leu-21 for a cysteine residue, which was apparently lost after mPEG-MAL conjugation. Although LyeTx I-bPEG exhibited a lower membrane affinity and revealed a partial loss of antimicrobial activity compared with LyeTx I-b, the structural and biophysical studies suggest no significant differences in the mode of the peptide-membrane interaction after PEGylation. Allied to these observations, the reduced cytotoxicity to VERO cells and improved proteolytic resistance observed after PEGylation demonstrate the great biotechnological potential of LyeTx I-bPEG for use as an antimicrobial agent.

## Data Availability

The raw data supporting the conclusion of this article will be made available by the authors, without undue reservation.
