# Supplementary material for: PEGylation of the antimicrobial peptide LyeTx I-b maintains structure-related biological properties and improves selectivity
Source: Front Mol Biosci. 2022 Oct 13;9:1001508. doi: 10.3389/fmolb.2022.1001508 (PMC9611540; doi:10.3389/fmolb.2022.1001508)
Supplement: Supplementary file 1 [file DataSheet1.PDF]

Supplementary Material

1 Supplementary Figures

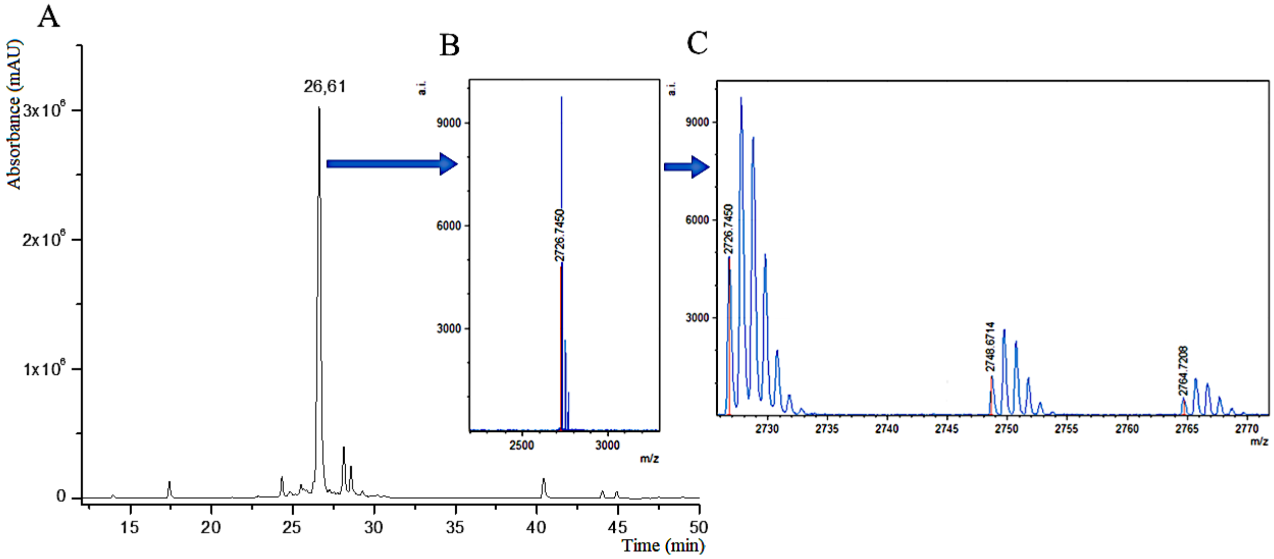

Figure S1. (A) LyeTx I-b$_{cys}$ purification chromatogram. The *y* axis represents the absorbance in mAU measured at the wavelength at 214 nm and the *x* axis, the time in minutes. Semi-preparative column Phenomenex C18, 10 µm, 250mm, 10mm. Mobile phase TFA in water 0.1 % (v/v) (phase A) and TFA 0.08 % (v/v) in acetonitrile (phase B). Chromatographic peak with elution time of LyeTx I-bPEG in 26.61 minutes. (B) Mass spectrum of the peak in 26.31 minutes showing LyeTx I-b$_{cys}$*m/z* of 2 726.7450 and (C) expansion of the mass spectrum showing the isotopic distributions of LyeTx I-b$_{cys}$. MALDI-TOF-MS with pepmix method (up to 4kDa). The *y* axis absorbance in a.i and *x* axis the charge mass in *m*/*z*.

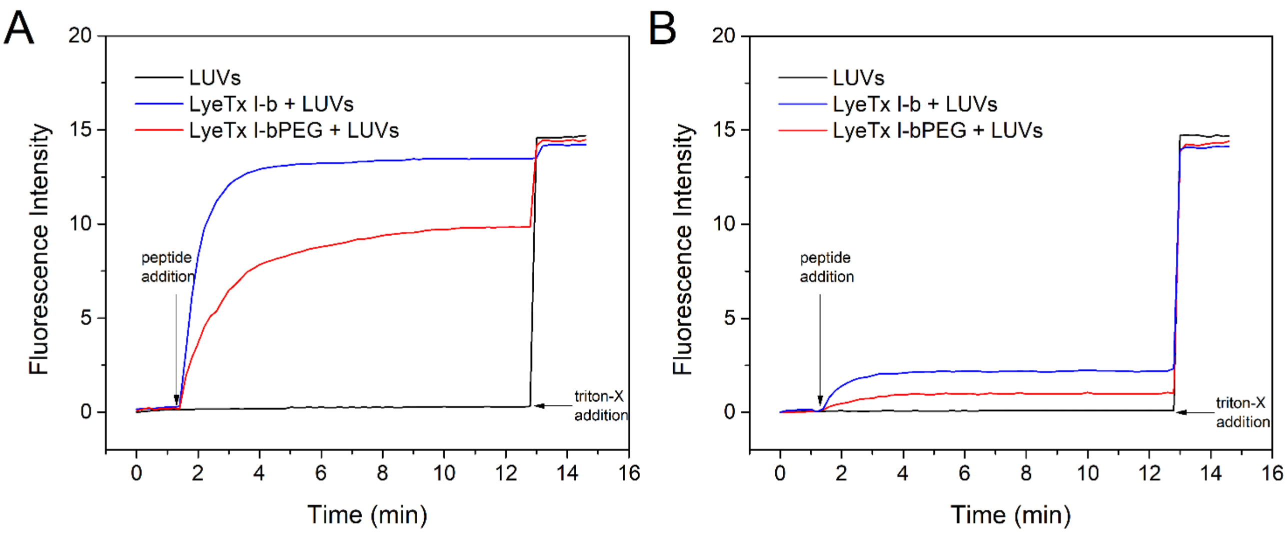

Figure S2. Release of calcein encapsulated in (**A**) 25 mM POPC:POPG LUVs (3:1 mol:mol) and (**B**) 25 mM POPC LUVs at 25 °C, induced by 16 μg/mL of LyeTx I-b (blue lines) and LyeTx I-bPEG (red lines).

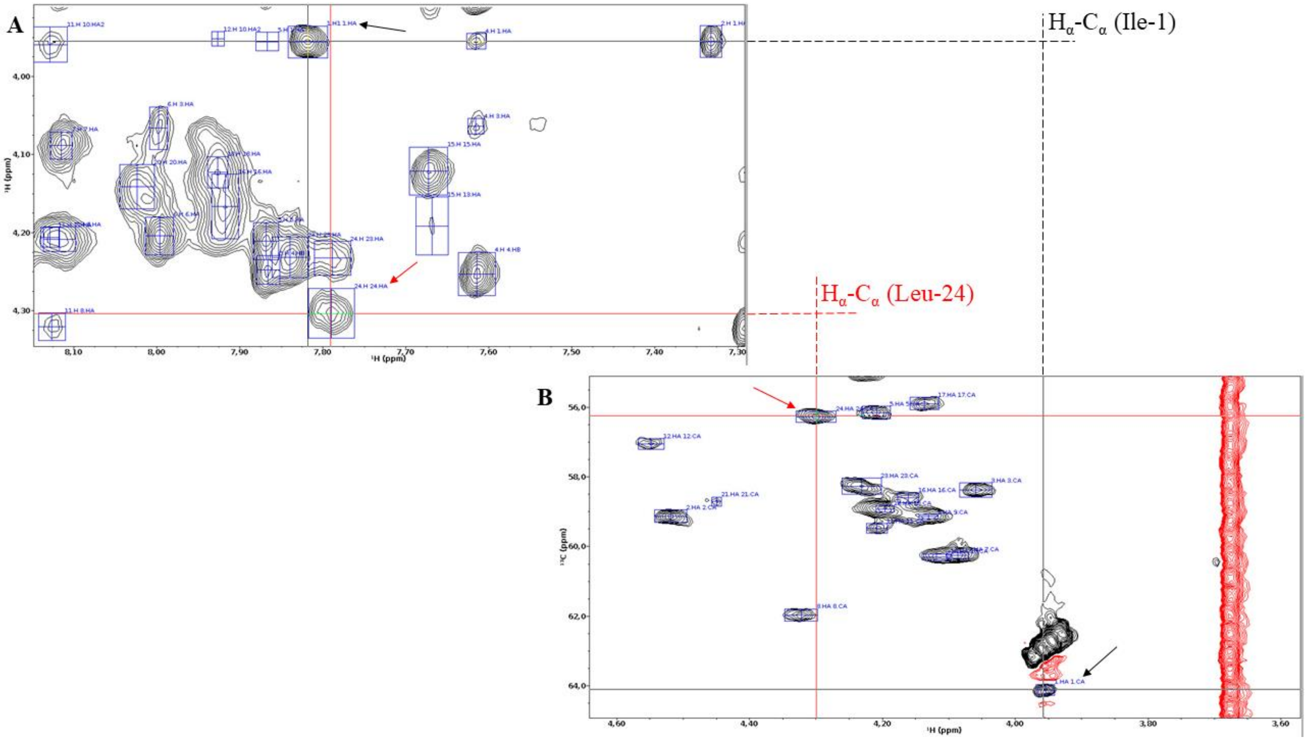

Figure S3. Partial (**A**) NOESY and (**B**) ^{1}H-^{13}C HSQC contour maps of LyeTx I-bPEG showing the Hα-Cα correlations for residues (red lines) Leu-24 and (black lines) Ile-1.

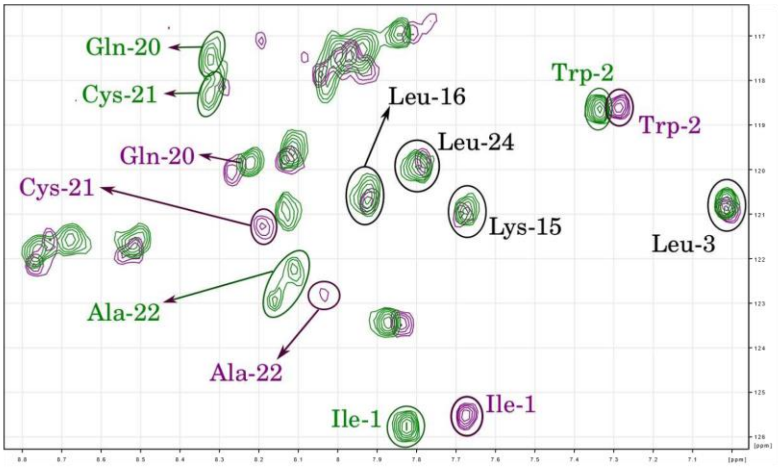

Figure S4. Superposition of LyeTx I-b$_{cys}$ (bordeau) and LyeTx I-bPEG (green) ^{1}H-^{15}N HSQC contour maps. Correlations highlighted from residues Gln-20 up to Ala-22 show a substantial chemical shift difference between LyeTx I-b$_{cys}$ and LyeTx I-bPEG, while residues that are far from the mPEG-MAL conjugation site, such as Leu-16, Leu-24, Lys-15 and Leu-3 (highlighted in black) have a similar chemical shift value between both peptides.

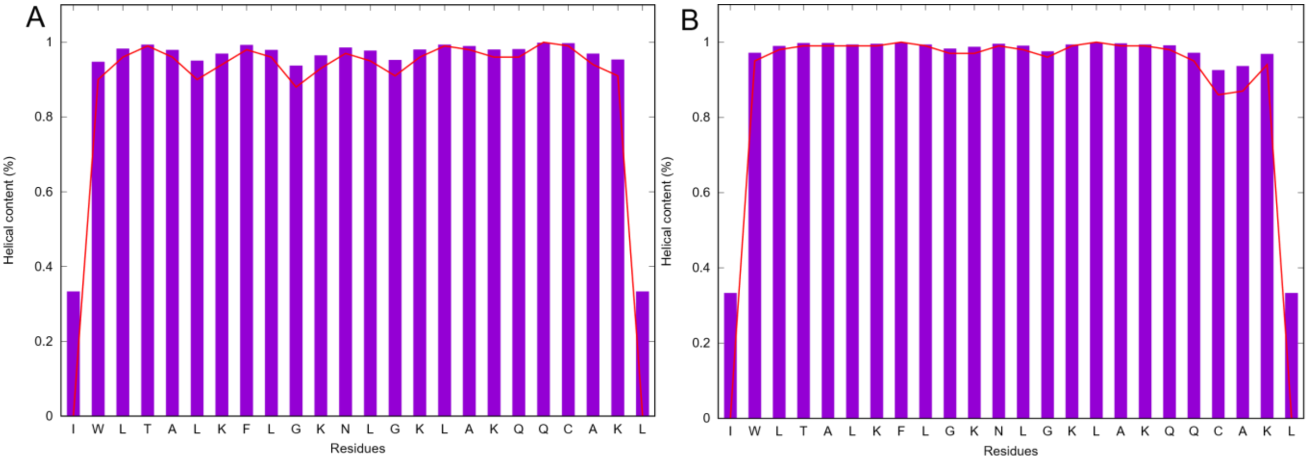

Figure S5. Neural-network-predicted helical content of each amino acid residue (purple bars) and their respective confidence values (red line) for (A) LyeTx I-b$_{cys}$ and (B) for LyeTx I-bPEG, as calculated by TALOS+ (Shen et al., 2009).

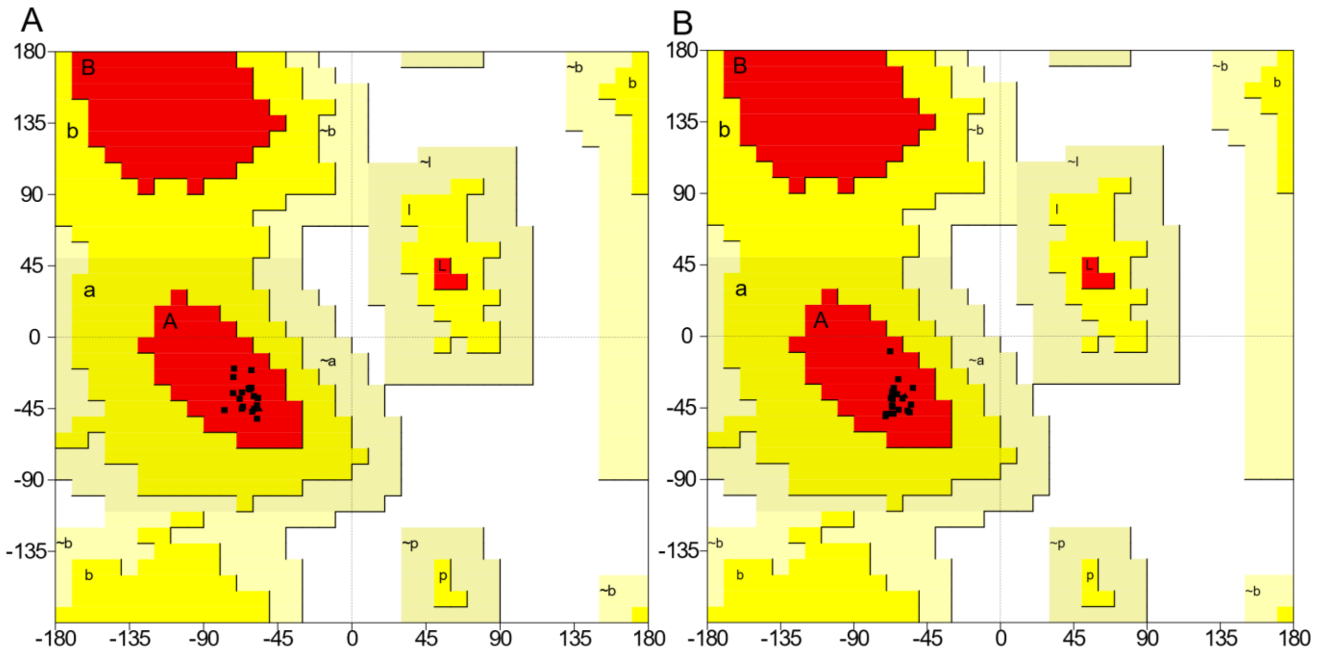

Figure S6. Ramachandran plots for the most stable calculated structure of (A) LyeTx I-b$_{cys}$ and (B) LyeTx I-bPEG.

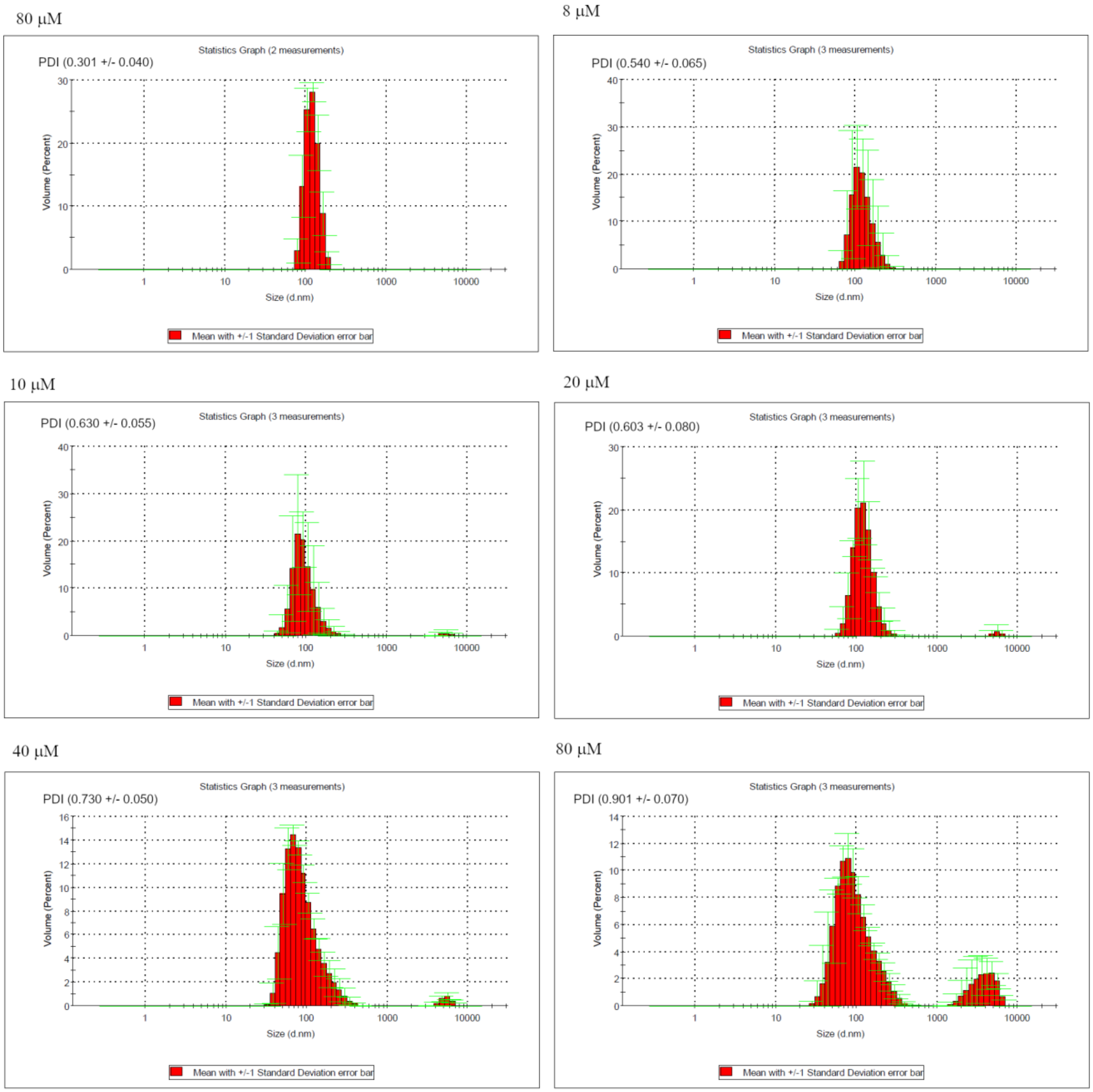

Figure S7. Polydispersity Index (PDI) values for LyeTx I-bPEG obtained at peptide concentrations ranging from 8 to 80 µM in the presence of POPC:POPG 3:1 (mol:mol) LUVs.

2 Supplementary Tables

Table S1. Comparison between chemical parameters of LyeTx I-b and LyeTx I-b_{cys}

	LyeTx I-b	LyeTx I-b_{cys}
Sequence	IWLTALKFLGKNLGKLAKQQ**L**AKL	IWLTALKFLGKNLGKLAKQQ**C**AKL

Mass (g/mol)	2737.4	2727.4
Molecular Formula	$C_{131}H_{222}N_{34}O_{29}$	$C_{128}H_{216}N_{34}O_{29}S$
C-terminus	$-NH_2$	$-NH_2$
N-terminus	$-CH_3CO$	$-CH_3CO$
Molar attenuation coefficient	5500	5500

Table S2. Comparison of RMSD values for all residues and helical segments of LyeTx I (Santos et al., 2010), LyeTx I-b (Reis et al., 2018), LyeTx I-b$_{cys}$ and LyeTx I-bPEG. Data was obtained by structural manipulation and visualization in MOLMOL(Koradi et al., 1996). Helical segments for LyeTx I comprised residues Thr-4 up to Leu-25 while for LyeTx I-b, LyeTx I-b$_{cys}$ and LyeTx I-bPEG comprised residues Trp-2 up to Lys-23.

	**LyeTx I**	**LyeTx I-b**	**LyeTx I-b$_{cys}$**	**LyeTx I-bPEG**
**RMSD (Å) – all residues**				
Backbone	0.99 ± 0.30	0.46± 0.18	0.85± 0.25	0.71± 0.22
Backbone and heavy atoms	1.97± 0.41	0.98± 0.21	1.62± 0.30	1.45± 0.25
**RMSD (Å) – helical segment**				
Backbone	0.72± 0.18	0.37± 0.16	0.72± 0.22	0.59± 0.19
Backbone and heavy atoms	1.47± 0.26	0.89 ± 0.21	1.49± 0.28	1.29± 0.24

3 References

Koradi, R., Billeter, M., and Wüthrich, K. (1996). MOLMOL: A program for display and analysis of macromolecular structures. *Journal of Molecular Graphics* 14, 51–55.

Reis, P. V. M., Boff, D., Verly, R. M., Melo-Braga, M. N., Cortés, M. E., Santos, D. M., et al. (2018). LyeTxI-b, a synthetic peptide derived from Lycosa erythrognata spider venom, shows potent antibiotic activity in vitro and in vivo. *Frontiers in Microbiology* 9, 1–12.

Santos, D. M., Verly, R. M., Piló-Veloso D.and de Maria, M., de Carvalho, M. A. R., Cisalpino, P. S., Soares, B. M., et al. (2010). LyeTx I, a potent antimicrobial peptide from the venom of the spider Lycosa erythrognata. *Amino Acids* 39, 135–144.

Shen, Y., Delaglio, F., Cornilescu, G., and Bax, A. (2009). TALOS+: a hybrid method for predicting protein backbone torsion angles from NMR chemical shifts. *Journal of Biomolecular NMR* 44, 213–223.
